# Expanding Access to Living Donor Kidney Transplants Through Social Networks

**DOI:** 10.1016/j.xkme.2023.100654

**Published:** 2023-05-06

**Authors:** Teri Browne, Julisa Tindall

**Affiliations:** College of Social Work, University of South Carolina, Columbia, SC

Related article, ●●●

The ideal treatment for individuals with kidney failure is kidney transplantation, with living donor kidney transplants offering the best outcomes and increasing the number of organs available for transplant.^1^^,^^2^ Accordingly, federal policies, such as the Centers for Medicare and Medicaid Services’ Conditions for Coverage,^3^ Advancing American Kidney Health Initiative,^4^ and End-Stage Renal Disease Quality Incentive Program,^5^ promote kidney transplantation and recommend that transplant centers and dialysis facilities work together to increase kidney transplants.

Targeted efforts to increase transplant parity are particularly needed because racial disparities in kidney transplants persist, including in living donation kidney transplants.^6^, ^7^, ^8^, ^9^ Contributors to kidney transplant parity are multifaceted^7^^,^^10^^,^^11^ and include social and structural determinants of health, such as social networks. In this issue of *Kidney Medicine*, Gillespie et al^12^ provided further insights into specific ways in which social networks can impact the outcomes of living donor kidney transplant. The authors identified how many hemodialysis patients’ network members offered to be kidney donors, whether these offers were from core or peripheral network members, and whose offers they accepted.

Social networks are relationships that individuals have with other people and are linked to health outcomes.^13^ Social networks can play an essential role in helping people successfully navigate kidney transplantation,^14^, ^15^, ^16^, ^17^, ^18^ with social network members influencing attitudes toward transplantation and helping with the transplant process. This study builds on the authors’ previous work, which suggests that a larger social network size can increase living donor requests,^15^ and expands this line of inquiry by examining the interconnectedness of dialysis patients’ social network members. Research on the role of social network attributes offers an innovative approach to inform much-needed interventions that can increase living donor kidney transplants and transplant parity.

As the authors noted, most living donors come from dialysis patients’ social networks. Currently, there is limited research that examined the availability of social network member donation offers and dialysis patients’ decisions to accept these offers. Understanding these processes is a critical first step toward informing future research and novel interventions that can promote living donor kidney transplants.

The authors administered a cross-sectional social network survey to 106 hemodialysis patients, examining the attributes of patient social networks, living donor offers, and patient decisions to accept such offers. The authors categorized social networks into core and peripheral members using egocentric network analysis. Core social network members represent a close connection, such as partners and family members, with whom patients discuss important matters. Core social network members also frequently know and have connections with one another. Peripheral social network members have fewer relationships with others in a patient’s social network, and patients are less likely to discuss important matters with these individuals.

Poisson regression models evaluated the relationship between social network attributes and kidney donor offers. Logistic regression models determined the associations between social network factors and patient acceptance of donation offers. The study respondents had a mean age of 60 years, 45% were women, and 75% identified as Black. Patients with more extensive social networks received more offers of kidney donations. In this study, 52% of the respondents received at least 1 living donor offer, and 42% of these offers were from peripheral network members. The authors concluded that dialysis patients with larger social networks comprising more peripheral members received more donation offers. Importantly, patients were 3.6 times more likely to accept donation offers from peripheral social network members than from core members.

The article by Gillespie et al^12^ provides a key insight into the decision-making process of dialysis patients related to living kidney donor offers. These findings suggest that peripheral social network members play a significant role in this process and are a significant source of kidney donor offers. Dialysis patients in this study were also more likely to accept these offers if they came from peripheral social network members.

The authors acknowledged several limitations of this study. This study involved only 106 dialysis patients; however, social network data analysis is an intensive process, and this analysis included 504 social network members. The study respondents came from only 2 dialysis units in 1 geographic area, and similar research is needed in other areas. This research relied only on patient reports about their social network members and donation offers; however, this is common in social network research. The respondents’ and network members’ knowledge of kidney transplant was not assessed; future research can explore these variables and examine their relationship with transplant success. Longitudinal research is also needed to better examine the impact of social networks on the actual receipt of a transplant.

A critical finding of this study is that despite over half of the respondents receiving a living donor offer and almost half of those offers being accepted, only 25% of patients with accepted offers were successfully evaluated for an actual transplant. This finding suggests that identifying facilitators to living donor kidney transplant offers is insufficient to help patients undergo transplants (as the authors acknowledged). Even with offers of living donation, this study adds to the robust literature suggesting that dialysis patients, especially Black patients, need extra help to complete all steps required for kidney transplant success.^11^^,^^18^^,^^19^ Efforts to examine the reasons and solutions for the disconnect between patient transplant interest and kidney donation offers and transplantation success must continue to address disparities in kidney transplantation in our country.

This article presents innovative approaches to examine living donor kidney transplantation and highlights an understudied contributor to transplant access. These findings can inform interventions that are prioritized across the country to promote kidney transplants among people receiving dialysis. Peripheral social network members may be important targets of these interventions. Efforts to explore potential living donors could include greater attention to these members, in addition to close social network members such as partners, close family, and friends. Gillespie et al^12^ provided suggestions for social network assessments that could help facilitate such efforts. Continued work in this area is needed to ensure that such assessments are feasible for use by dialysis centers.

This study also highlights the importance of dialysis teams considering social support network attributes as a part of patient care in planning for kidney transplantation. Dialysis centers are essential in promoting and facilitating kidney transplants, including living donor transplants.^20^, ^21^, ^22^ Dialysis team members should provide patients with education, advocacy, and assistance needed to help patients succeed in navigating the steps toward living donor kidney transplants.

Overall, the study by Gillespie et al^12^ provides innovative insights into the decision-making process of dialysis patients related to living kidney donor offers and the role of social networks in living donor kidney offers. Increasing transplant rates requires the expansion of living donor kidney transplants and involves a multifaceted approach that addresses the social and structural factors that contribute to transplant success. Peripheral social network members are important targets of these interventions, and efforts to explore potential living donors could include attention to these members, in addition to partners as well as close family and friends.

## References

[1] Wolfe R.A., Ashby V.B., Milford E.L. (1999). Comparison of mortality in all patients on dialysis, patients on dialysis awaiting transplantation, and recipients of a first cadaveric transplant. N Engl J Med.

[2] de Groot I.B., Veen J.I.E., van der Boog P.J.M. (2013). Difference in quality of life, fatigue and societal participation between living and deceased donor kidney transplant recipients. Clin Transplant.

[3] Centers for Medicare and Medicaid Services (CMS), HHS (2008). Medicare and Medicaid programs; conditions for coverage for end-stage renal disease facilities. Final rule. Fed Regist.

[4] Advancing American Kidney Health Initiative Federal Register. https://www.federalregister.gov/documents/2019/07/15/2019-15159/advancing-american-kidney-health.

[5] ESRD quality incentive program. Centers for Medicare and Medicaid Services. https://www.cms.gov/Medicare/Quality-Initiatives-Patient-Assessment-Instruments/ESRDQIP.

[6] Ernst Z., Wilson A., Peña A., Love M., Moore T., Vassar M. (2023). Factors associated with health inequities in access to kidney transplantation in the USA: a scoping review. Transplant Rev.

[7] Husain S.A., King K.L., Adler J.T., Mohan S. (2022). Racial disparities in living donor kidney transplantation in the United States. Clin Transplant.

[8] Taber D.J., Gebregziabher M., Hunt K.J. (2016). Twenty years of evolving trends in racial disparities for adult kidney transplant recipients. Kidney Int.

[9] Wesselman H., Ford C.G., Leyva Y. (2021). Social determinants of health and race disparities in kidney transplant. Clin J Am Soc Nephrol.

[10] Purnell T.S., Xu P., Leca N., Hall Y.N. (2013). Racial differences in determinants of live donor kidney transplantation in the United States. Am J Transplant.

[11] Park C., Jones M.M., Kaplan S. (2022). A scoping review of inequities in access to organ transplant in the United States. Int J Equity Health.

[12] Gillespie A, Daw J, Brown R, et al. Dialysis patients’ social networks and living donation offers. *Kidney Med*. Preprint posted online April 15, 2023. https://doi.org/10.1016/j.xkme.2023.100640 10.1016/j.xkme.2023.100640 PMC10206208 37235041

[13] Ladin K., Hanto D.W. (2010). Understanding disparities in transplantation: do social networks provide the missing clue?. Am J Transplant.

[14] Arthur T. (2002). The role of social networks: a novel hypothesis to explain the phenomenon of racial disparity in kidney transplantation. Am J Kidney Dis.

[15] Gillespie A., Gardiner H.M., Fink E.L., Reese P.P., Gadegbeku C.A., Obradovic Z. (2020). Does sex, race, and the size of a kidney transplant candidate’s social network affect the number of living donor requests? A multicenter social network analysis of patients on the kidney transplant waitlist. Transplantation.

[16] Browne T. (2011). The relationship between social networks and pathways to kidney transplant parity: evidence from black Americans in Chicago. Soc Sci Med.

[17] Aljurbua R., Gillespie A., Obradovic Z. (2022). The company we keep. Using hemodialysis social network data to classify patients’ kidney transplant attitudes with machine learning algorithms. BMC Nephrol.

[18] Kumar K., Tonascia J.M., Muzaale A.D. (2018). Racial differences in completion of the living kidney donor evaluation process. Clin Transplant.

[19] Patzer R.E., Paul S., Plantinga L. (2017). A randomized trial to reduce disparities in referral for transplant evaluation. J Am Soc Nephrol.

[20] Patzer R.E., Pastan S.O. (2020). Policies to promote timely referral for kidney transplantation. Semin Dial.

[21] Patzer R.E., Plantinga L., Krisher J., Pastan S.O. (2014). Dialysis facility and network factors associated with low kidney transplantation rates among United States dialysis facilities. Am J Transplant.

[22] Browne T., Amamoo A., Patzer R.E. (2016). Everybody needs a cheerleader to get a kidney transplant: a qualitative study of the patient barriers and facilitators to kidney transplantation in the Southeastern United States. BMC Nephrol.

